# Self-Organization of Muscle Cell Structure and Function

**DOI:** 10.1371/journal.pcbi.1001088

**Published:** 2011-02-24

**Authors:** Anna Grosberg, Po-Ling Kuo, Chin-Lin Guo, Nicholas A. Geisse, Mark-Anthony Bray, William J. Adams, Sean P. Sheehy, Kevin Kit Parker

**Affiliations:** Disease Biophysics Group, Wyss Institute for Biologically Inspired Engineering, School of Engineering and Applied Sciences, Harvard University, Cambridge, Massachusetts, United States of America; University of Auckland, New Zealand

## Abstract

The organization of muscle is the product of functional adaptation over several length scales spanning from the sarcomere to the muscle bundle. One possible strategy for solving this multiscale coupling problem is to physically constrain the muscle cells in microenvironments that potentiate the organization of their intracellular space. We hypothesized that boundary conditions in the extracellular space potentiate the organization of cytoskeletal scaffolds for directed sarcomeregenesis. We developed a quantitative model of how the cytoskeleton of neonatal rat ventricular myocytes organizes with respect to geometric cues in the extracellular matrix. Numerical results and *in vitro* assays to control myocyte shape indicated that distinct cytoskeletal architectures arise from two temporally-ordered, organizational processes: the interaction between actin fibers, premyofibrils and focal adhesions, as well as cooperative alignment and parallel bundling of nascent myofibrils. Our results suggest that a hierarchy of mechanisms regulate the self-organization of the contractile cytoskeleton and that a positive feedback loop is responsible for initiating the break in symmetry, potentiated by extracellular boundary conditions, is required to polarize the contractile cytoskeleton.

## Author Summary

How muscle is organized impacts its function. However, understanding how muscle organizes is challenging, as the process occurs over several length scales. We approach this multiscale coupling problem by constraining the overall shapes of muscle cells to indirectly control the organization of their intracellular space. We hypothesized the cellular boundary conditions direct the organization of cytoskeletal scaffolds. We developed a model of how the cytoskeleton of cardiomyocytes organizes with respect to boundary cues. Our computational and experimental results to control myocyte shape indicated that distinct muscle architectures arise from two main organizational mechanisms: the interaction between actin fibers, premyofibrils and focal adhesions, as well as cooperative alignment and parallel bundling of more mature myofibrils. We show that a hierarchy of processes regulate the self-organization of cardiomyocytes. Our results suggest that a symmetry break, due to the boundary conditions imposed on the cell, is responsible for polarization of the contractile cytoskeletal organization.

## Introduction

During biological development, evolving forms are marked by distinct functionalities. An interesting example is the organization of myofibrils in striated muscle cells. As the myocyte matures, the myofibrils are rearranged from an irregularly dispersed pattern into tightly organized bundles spanning the length, rather than the width, of the cell [1]. Although assembly of the myofibril from its molecular constituents has been extensively investigated [2], [3], [4], how myofibrils build this specialized architecture and its functional consequences remains unanswered. This is important because changes in muscle structure accompany not only morphogenesis, but also pathogenesis [5], [6].

Myofibrils mature in a force-dependent manner [7], [8], [9], suggesting that the contractility of a cell may play an important role in polarizing the myofibrillar network. This has been shown in nonmuscle cells where the cytoskeletal architecture within a geometrically-defined microcompartment becomes polarized with increasing tractional forces [10], [11]. Thus, we hypothesized that geometric cues in the extracellular matrix (ECM) can organize the intracellular architecture and potentiate directed myofibrillogenesis. Because of the difficulty in identifying *de novo* sarcomeres in primary harvest muscle cells in culture, one strategy for studying myofibrillogenesis is to coax the disassembly and reassembly of myofibrils by forcing myocytes to assume shapes that are not commonly observed *in vivo* using engineered substrates *in vitro* [10], [11]. To guide these experiments, we developed a computational model of myofibrillar patterning to show the sensitivity of the intracellular architecture to the extracellular space. With these tools, we sought to understand the critical events in the global assembly and organization of the contractile apparatus in cardiac myocytes. By comparing experimental results with our computational model, we were able to elucidate the role of maturing myofibrils, their parallel coupling, and their functional attachment to the focal adhesion assembly and how these processes are guided spatially by the boundary conditions imposed on the cell. After determining the roles of these parameters in myofibrillogenesis, we then expanded our model to test the functional implications of these architectures. We developed a novel method for micropatterning on soft substrates and were able to engineer myocyte shape on substrates that would allow us to measure the contractility of these artificial shapes and compare them with the model results. Together, these results suggest that the self-assembly and -organization of the contractile apparatus is facilitated by a symmetry-breaking event that is potentiated by either a geometric cue in the extracellular space or a random event in the intracellular space.

## Results

### Qualitative Description of the Model

**Figure 1:**
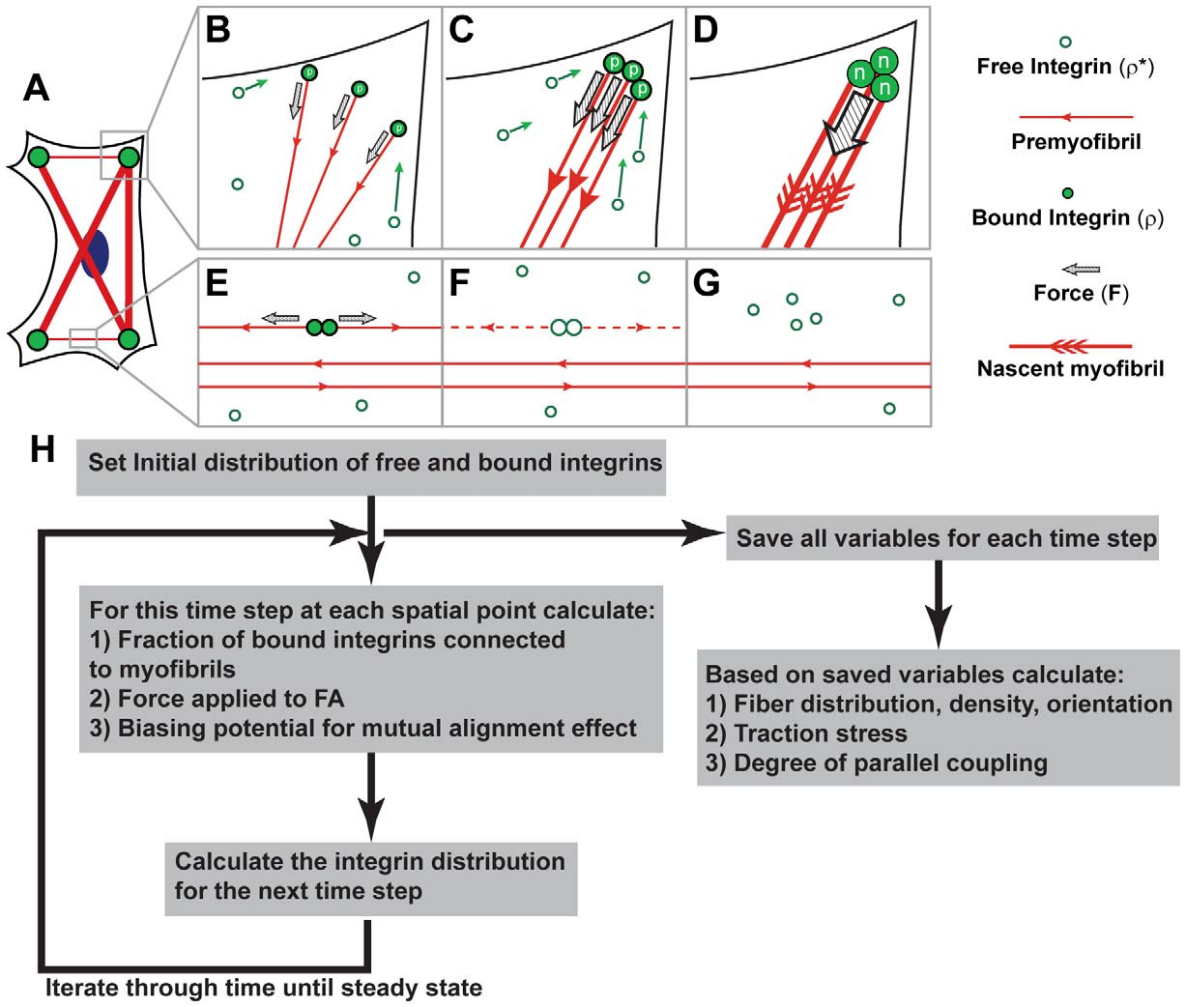
Schematic representation of myofibril reorganization in a 2D myocyte. (A) red: actin; blue: nucleus; green: FAs. The FAs can spread throughout the ECM island (outlined by solid black island). (B) Net force (**F**) exerted on bound integrins, as determined by the sum of all forces exerted by the anchoring premyofibril vectors, recruits free integrins and promotes growth of FAs. For the purposes of modeling the bound integrins connected to premyofibrils are labeled *ρ_p_*(**r**). (C) Continued recruitment of free integrins to the growing FA at the cellular corners is associated with enhanced bundling of the premyofibrils and subsequently increased traction. (D) Built upon the premyofibrillar network, the nascent myofibrils align in parallel and develop into a fully organized bundle, further amplifying local force to result in FA maturation. For the purposes of modeling the bound integrins connected to nascent myofibrils are labeled as *ρ_n_*(**r**). (E) Bound integrins with zero net force cannot recruit free integrin and are disassociated from the membrane, leading to release of the attached fiber (F). Consequently, contractile fibers on shorter axes (G) are less bundled than that following the longest diagonal of the cell. (H) Qualitative schematic of model implementation algorithm.

Our theoretical approach focuses on the interaction between the myofibril and the ECM, as well as adjacent myofibrils (Fig. 1). Inherent to our model are two key assumptions: 1) the force that the myofibrillar bundle exerts on the substrate is fiber length-dependent [12] and 2) adjacent myofibrils affect each other to facilitate lateral coupling, which is akin to them exerting torque on each other. We have modeled only the maturation of cytoskeletal structural elements responsible for contraction and integrin binding to the ECM. We define these components using coarse-grained variables that are experimentally observable. This eliminates the computational complexity required to model detailed molecular interactions and the effect of different protein isoforms. The nomenclature for the immature and mature versions of the myofibril vary with different qualitative models (reviewed by Sanger and colleagues [2]). Here we refer to the immature state as the premyofibril, and the quasi-mature state as the nascent myofibril [1], [13].

Our mathematical approach differs from others [14], [15] in that we incorporate focal adhesion (FA) kinetics, mutual alignment of adjacent contractile fibers, and the dependence of contractile forces on fiber length [16]. The variables used in our approach are: (1) the density of bound and unbound integrin, 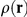 and 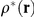, respectively; with the bound integrins connected to premyofibrils and nascent myofibrils labeled as 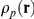 and 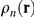, respectively; (2) the net force exerted on the bound integrin, 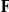; (3) the local density, 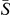, orientation, 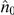, and the orientational order parameter, 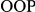, of the premyofibril network and the nascent myofibril network; and (4) the resultant 2D stress field exerted by the cell on the substrate, **T**. Previously, we reported [17] that when cardiac myocytes are constrained on 2D islands, their vertical dimension, orthogonal to the plane of the culture surface, is uncontrolled. In that study, we reported that myofibrils are predominantly located under the nucleus, in a plane parallel to the culture surface. However, as that study also showed, several layers of myofibrils may be present, and the nucleus and microtubule organizing center may represent an obstacle to a symmetrical array of myofibrils in the thicker regions of the cell. Our model and analysis is restricted to the 2D intracellular plane closest to the culture surface. Instead of solving the steady state for all of the variables, we numerically simulated their spatiotemporal profiles. This allows us to trace the effect of local symmetry-breaking events such as the mutual alignment of fibers on myofibrillar patterning, which cannot be easily predicted by conventional steady-state analysis. The local symmetry-breaking event may result from a static cue or a transient perturbation.

In our simulation, we began with randomly distributed densities of the unbound integrin, unless fitting parameters, in which case we examined several sets of initial conditions. The unbound integrin can initially become bound through a random process, with the rate proportional to its local concentration. The fraction of bound integrins connected to the fibrils is modeled as an adsorption process, and is calculated using the Langmuir isotherm. The force exerted between FAs is assumed to be proportional to the product of fiber connections at each site [16]. The net force at a local FA is computed by integrating the tension contributed by all connected contractile elements (Fig. 1A). The net force governs the growth rate of local FAs, which in turn modulates the premyofibril network [18], [19]. The assembly of FAs and the bundling of its associated fibers is coupled by a positive feedback loop via forces exerted on the FA [16], [18]. As a consequence of the positive feedback, when the net force on a FA is not zero, both the FA and its associated fibers are structurally reinforced (Fig. 1B–D) [20]. If the net force is zero, the bound integrins will disassemble at each time step and disassociate the attached fibers (Fig. 1E–G) [18], [21]. As time lapses, the premyofibrils are converted to the nascent myofibrils. The local orientation of the nascent myofibril is primarily determined by the antecedent premyofibril network, but also can be modulated by adjacent myofibrils due to their lateral coupling [1], [22]. In some cell shapes, polarization of the myofibrillar array can only be achieved by the lateral alignment of adjacent myofibrils, which occurs at a much slower time scale than that of fiber assembly [1], [22]. The effect of the lateral coupling is modeled as a biasing potential field that distributes the free integrins, such that the nascent myofibrils are moved towards each other through the course of normal integrin recycling. To visualize the amount of parallel, or lateral, coupling of the fibers, we define a variable, *ψ*, which varies from zero for no local coupling, to unity for the maximal local coupling. The model's calculations are ordered as depicted in Fig. 1H.

### Model versus Experiment: The Architecture of a Stair-Shaped Myocyte

**Figure 2:**
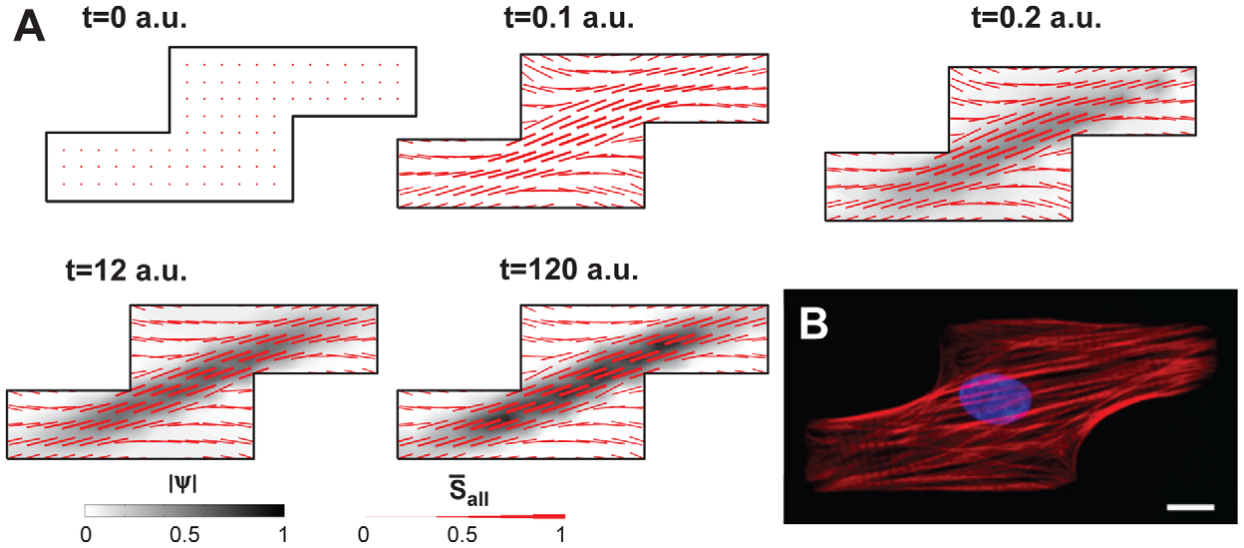
Simulated dynamics of myofibril organization and immunostaining of actin alignment. (A) Simulated results for the dynamic profile of myofibril organization in a stair-step-shaped myocyte. Red lines represent the myofibrils, with thicker lines representing regions of denser myofibrils. The grey color scale represents the amount of local parallel coupling of the nascent myofibrils; color values are in arbitrary units normalized to the highest values. As we start with a random distribution of free integrins, initially there were no fibers. The geometrical symmetry break in the stair-cell is so strong that for random initial conditions the fibers generally align with the major diagonal as soon as they are formed. However, nascent myofibrils become latterly coupled throughout the cell as evident by the diffuse grey shading at 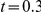. As time elapsed, the nascent myofibrils reorganized and oriented themselves along the longest cellular diagonal, and coupled to each other greatly increasing parallel coupling. The steady state fiber organization matches the experimental results. (B) Immunostaining of the actin network from a myocyte with similar shape agrees with the numerical prediction; scale bar: 10 µm.

To fit the parameters of the computational model, we chose an uncommon cell shape, a stair-shaped myocyte, that we could model computationally *in silico* and repeatably *in vitro* with cell engineering techniques (Fig. S1). The parameters were fit on a variety of initial conditions (Fig. S2) such that the steady state results were the same for each. In Fig. 2A we show the temporal results for an initial condition with a random distribution of free integrins. Initially, there are no fibers in the cell, as no integrins are bound (Fig. 2A 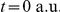). The geometrical symmetry of the stair-shape cell potentiates the initial appearance of fibers predominantly along the diagonal. As the fibers form, the fiber density is mostly uniform throughout the cell, as evident from the line segment thickness (Fig. 2A 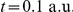). When the nascent myofibrils form and begin to laterally couple, they are distributed diffusely within the cell (Fig. 2A 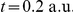). As time progresses, the positive feedback increases, i.e. greater number of fibers produces a greater force which drives the clustering of bound integrins and fibers. As a result, the myofibrils achieve a distribution very similar to the steady state (Fig. 2A 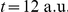). For the rest of the simulation the nascent myofibrils mutually align and exhibit greater degrees of parallel coupling (Fig. 2A 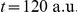). Myocytes were cultured on stair-step shaped islands for three days and then stained against actin filaments (Fig. 2B). At equilibrium, most nascent myofibrils are coupled and aligned with the major diagonal, as shown experimentally in Fig. 2B and in simulation (Fig. 2A 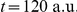). The parallel coupling of the nascent myofibrils emerges later in the simulation, as suggested by previous reports [1], [21], [22], [23]. In summary, the simulated dynamics visualized for nascent myofibril bundling and realignment show that well-aligned myofibrils first occurred in the center of the cell, followed the longest diagonal, and recruited additional adjacent fibers to form a bundled, parallel arrangement.

### Model versus Experiment: Heterogeneous and Homogeneous Boundary Conditions

**Figure 3:**
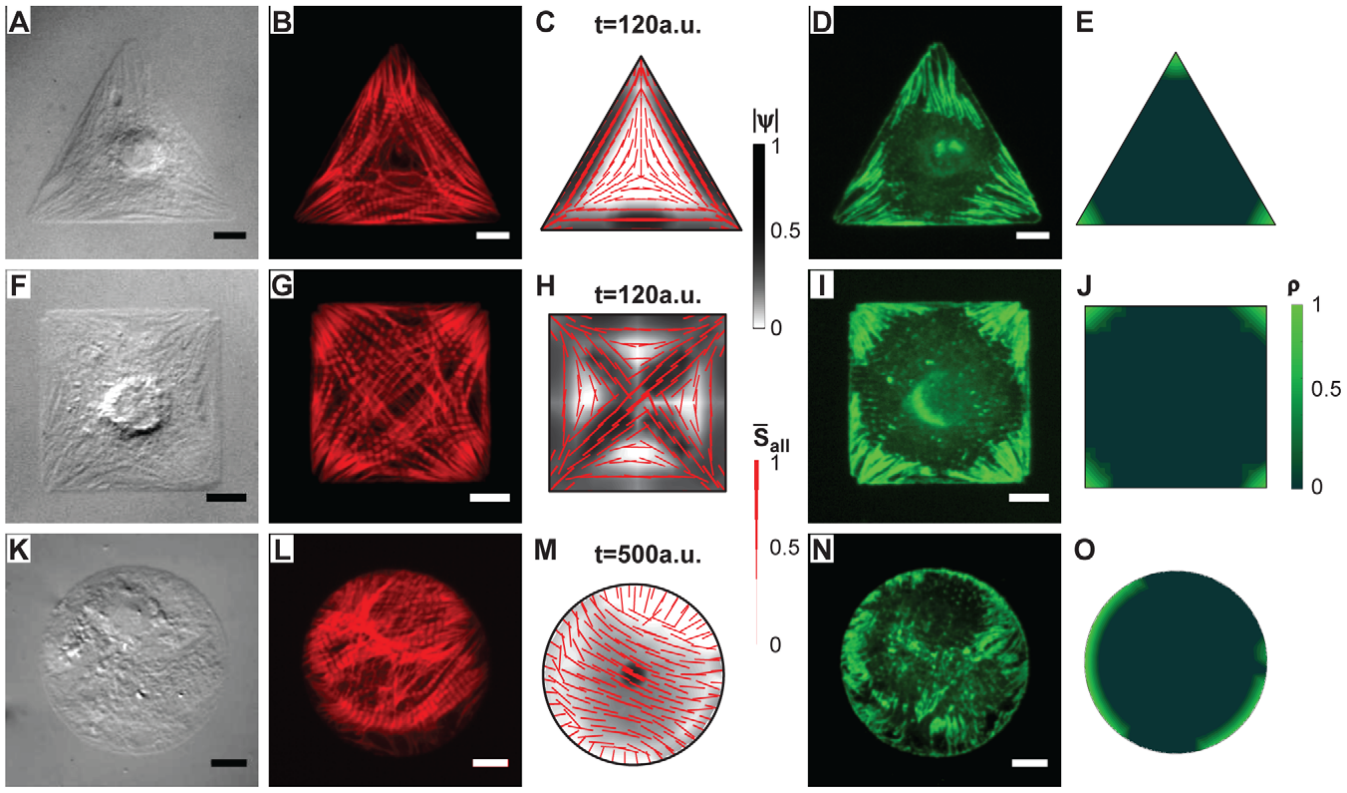
Experimental images and model depictions of organization of actin and FAs. *First column:* DIC images of micropatterned triangular (A), square (F), and circular (K) myocytes. *Second column:* Immunostained actin in triangular (B) and square (G) myocytes followed the longest cellular dimension, while actin fibers in the circular myocyte (L) primarily oriented on the 2 to 8 o'clock axis. *Third column:* Predicted myofibrillar pattern of triangular (C), square (H), and circular (M) myocytes agrees with experimental results. The steady state of the circular cell occurred slower than that of the triangular and square cells. The thickness of the lines is proportional to the myofibril density 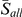. The grey color scale represents myofibril bundling, i.e. degree of parallel coupling 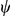. *Fourth column:* Immunostained vinculin of triangular (D) and square (I) myocytes was concentrated at cellular corners, while two opposing plaques of vinculin localized on the 2 to 8 o'clock axis in the circular (N) myocyte. *Fifth column:* Simulated FA density (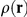) at steady state in triangular (E), square (J), and circular (O) cells was consistent with experimental results. The FA distribution in a circular myocyte (O) was expected to break the symmetry. Color values in simulated results are in arbitrary units scaled from 0 to 1; scale bars are 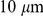.

To test our hypothesis, we examined the sensitivity of myocytes and our model to various cellular boundary conditions. We reasoned that when myocytes are constrained by a heterogeneous boundary curvature, triangles (Fig. 3A) and squares (Fig. 3F), the distinct geometrical cues at the cell boundaries would potentiate unique cytoskeletal architectures, but when cells are constrained by a homogeneous boundary curvature, a circle (Fig. 3K), there is no external cue to break the symmetry of the isotropic network. Thus, we examined two cases of the cell with heterogeneous curvature at the periphery: the square shaped cell, where the longest axes are on the diagonal, and the equilateral triangle shaped cell, where the long axes are along the cell periphery. We also tested cells with homogeneous boundary curvature: the circular shaped cell, in which no major axis is defined. To ensure that the observations resulted from geometric considerations alone, we used the same parameter values from the previous simulations.

Fluorescent staining of actin filaments in myocytes cultured on square and triangular ECM islands for 72 hrs revealed that polymerized actin fibers were densely arranged along the longest axes (Fig. 3). The fibers are regularly punctuated along their length, indicating the presence of sarcomeres (Fig. 3B, G). At steady state, modeled triangular and square cells displayed the same cytoskeletal arrangement as the *in vitro* results, with enhanced parallel bundling occurring along the longest axis of these cells (Fig. 3C, H). Fluorescent staining of vinculin revealed elongated FAs in the corners of the square and triangular cells that were oriented in parallel with their attached myofibrils (Fig. 3D, I). Numerical results revealed the same accumulation pattern of FAs, as indicated by the density of bound integrin located in the corners (Fig. 3E, J). The dynamics of the simulation results are depicted in Fig. 3C, E, H, and J and Video S1, S2, S3, and S4. As previously observed in the simulation shown in Fig. 2, the predominant orientation of the premyofibrils occurs quickly and the parallel bundling increased with time to further stabilize the myofibrillar architecture with respect to the geometric cues in the ECM. These data suggest that FAs localize and mature at the corners because the premyofibrils that align along the longest axes of the cell are the strongest by virtue of their greater propensity for parallel bundling and binding myosin motors [24], [25].

In contrast, myocytes cultured on circular ECM islands (Fig. 3K) for the same period of time have random myofibrillar architectures (Fig. 3L) [26], which is recapitulated in the model (Fig. 3M). Without an external cue to break the geometric symmetry, computer simulations suggest that myofibrillar polarity will emerge after a longer period of time, (almost five times as long as other shapes). Transient multi-pole patterns develop within cellular microcompartments (Video S5) and at equilibrium there is local bundling and nascent myofibril formation, but no overall cell organization (Fig. 3M, Video S5). *In vitro*, vinculin stains irregularly around the myocyte perimeter (Fig. 3N). *In silico*, after a similarly prolonged simulation, FAs appear as opposing bands along the cell periphery (Fig. 3O, Video S6). It is important to note that this patterning is due to a random, intercellular, symmetry-breaking event and that while the model will always converge, circular cells both *in silico* and *in vitro*, after 2–3 days in culture, often display irrepeatable cytoskeletal structures. Together, the simulation and experimental results summarized in Fig. 3 suggest that the orientation of the premyofibrillar network is regulated by ECM cues. These cues promote stabilization of the network and FAs, facilitating parallel bundling of the nascent myofibrils. Furthermore, our model predicted that the polarized myofibrillar network has a preference to align along the longest axis of cells.

### Model versus Experiment: Contractility

**Figure 4:**
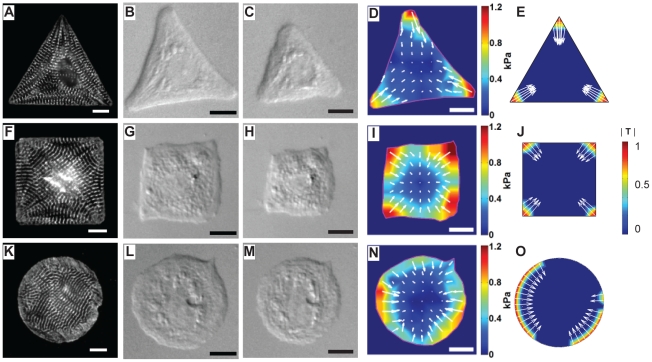
Sarcomeric structure, traction force at peak systole, and model predictions. *First column:* Sarcomeric 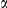-actinin immunofluorescence delineates the Z-lines in triangular (A), square (F) and circular (K) myocytes. Z-line orientation indicated that the axis of contraction was parallel to the longest axis of the cell. In the circular myocyte, most of the Z-lines aligned on the 1 to 7 o'clock axis with the dominant axis of contraction expected to follow the 4 to 10 o'clock direction. *Second column:* DIC images of micropatterned triangular (B), square (G), and circular (L) myocytes at full relaxation. *Third column:* DIC images at full contraction of the triangular (C), square (H), and circular (M) myocytes show the cells shortened about 24%, 18%, and 14% along the longest cell dimension, respectively. *Fourth column:* The contractile traction map of the triangular (D) and square (I) myocytes displayed high traction stresses at the cellular corners. The contraction map of the circular myocyte (N) indicated that the cell broke radial symmetry, with the principal axis of contraction along the 3 to 9 o'clock axis. *Fifth column:* Numerical results of predicted traction (**T**) of triangular (E), square (J), and circular (O) myocytes replicated experimental results. In the fourth and fifth columns, the color scale and arrows represent the magnitude and direction of traction, respectively. Color values in simulated results are in arbitrary units; scale bars are 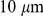.

Proper functioning of myocytes requires the correct myofibrillar configuration for coordinated contraction [5]. To correlate myofibrillar structure with contractile function, we investigated the spatial patterning of sarcomeric proteins and conducted traction force microscopy on the cultured myocytes. Fluorescent micrographs of myocytes immunostained against sarcomeric α-actinin revealed distinct myofibrillar patterning on ECM islands of heterogeneous boundary curvature (Fig. 4A, F). The sarcomeric Z-lines register in the internal angles of the corners of both the square and triangle and are perpendicular to the orientation of the actin fibers. To measure myocyte contractile stresses, we engineered ECM islands on soft substrates. When freshly harvested myocytes are cultured on these substrates, they remodel to assume the shape of the island in the same manner as they do on rigid substrates (Fig. 4B, G). Unlike myocytes cultured on the rigid substrates, myocytes on soft substrates do not contract isometrically and can be observed to shorten as in traditional assays of single myocyte contractility (Fig. 4C, H, Video S7 and S8). To visualize substrate deformation due to myocyte contraction, fluorescent beads were embedded in the substrate and bead movement was detected using high speed fluorescence microscopy. The nominal stress field exerted on the substrate due to systolic contraction, with the resting myocyte position defined as the reference state, was calculated from substrate deformation with the known substrate mechanical properties and assuming that the substrate is linearly elastic. In the videos (Videos S9 and S10), the substrate displacement vectors, as depicted by the white arrows, are directed inward during systole, indicating that the substrate is pulled towards the center of the myocyte by the shortening FA-anchored myofibrils. During diastole, they reversed direction as the elastic recoil of the myocyte pushed the substrate back to the rest position. The myocytes generate a unique contractile footprint that mimics the position of the FAs depicted in Fig. 3, with the highest systolic stresses exerted on the substrate at the corners of the myocyte (Fig. 4D, I). Note that even though the model does not differentiate between systolic and diastolic stresses, the experimental substrate stress field pattern matches the simulated results (Fig. 4E, J).

In myocytes of homogeneous boundary curvature, the myofibrillar patterns are not reproducible. However, structural coordination of the myofibrils on a preferential axis was observed, as evidenced by the well-demarcated Z-lines that continuously traversed the 1 to 7 o'clock axis in the circular myocyte shown in Fig. 4K. Similarly, the circular shaped myocytes cultured on soft substrates appear to shorten concentrically during contraction (Fig. 4L, M, Video S11), where a principal axis of shortening is apparent at peak systole but does not occur with the same spatial regularity of the square and triangular cells (Fig. 4N, Video S12), consistent with previous findings with nonmuscle cells [10]. Our model predicted a similar contractile signature (Fig. 4O), with the peak stresses coincident with the location of the widest FA bands observed in Fig. 3O. Thus, these data suggest that muscle cells depend on extracellular spatial cues to efficiently and functionally organize the myofibrils and contracion.

### Hierarchy of Organizing Strategies: Force-Length Dependence vs. Mutual Alignment

**Figure 5:**
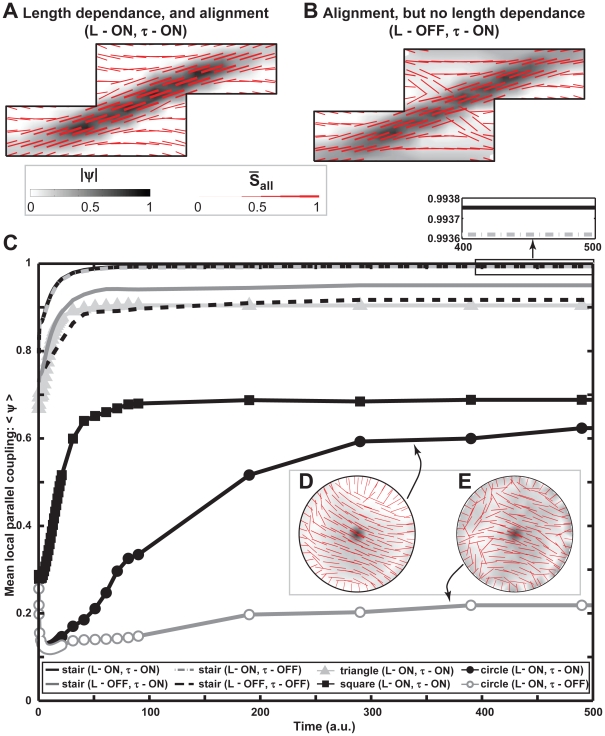
Testing model assumptions *in silico*. (A,B,D,E) Steady state results for different conditions tested *in silico*, where the red segments correspond to the direction of the all fibers, and the thickness of the red segments is proportional to the density of the fibers. The grey contour represents the degree of parallel coupling. Note that all the values were normalized by the maximum across all the conditions for ease of comparison between them. C) Plot of averaged (over the whole cell) degree of parallel coupling. Stair shape cell: fiber length-force independence, but mutual alignment –solid grey line; fiber length-force independence and no mutual alignment – dashed black line; fiber length-force dependence and mutual alignment - solid black line; no mutual alignment, but fiber length-force dependence - dash-dot grey line; the inset shows the difference in steady state values between the two latter cases. Triangle cell with both fiber length-force dependence and mutual alignment – grey line, triangular markers. Square cell with both fiber length-force dependence and mutual alignment – black line, square markers. Circular cell with fiber length-force dependence, with mutual aligment and no mutual aligment is shown as a black line with circular markers, and a grey line with empy circular markers, respectively. Comparing steady states for stair cells in (A) and (B) illustrates the necessity of fiber length-force dependence, while comparison of circular cells in (D) and (E) illustrates the necessity of mutual alignment of fibers.

We hypothized that a hierarchy of mechanisms may be responsible for myofibrillar organization. We reasoned that our model would allow us to determine which of the two model features, the fiber length-force dependence and parallel coupling of fibers, was dominant in organizing the myofibrillar architecture. We also reasoned that the nature of the cell boundaries may determine the sensitivity of the cell to these two mechanisms. To test this hypothesis, we ran simulations where these two features were either on, or turned off, within the cell. In the staire-shaped cell, we ran simulations where: 1) there is no mutual alignment mechanism but fiber contractility is fiber length-dependent (*L* = ON, *τ* = OFF, refer to Eq. (1) & (4)); 2) the fiber contractility is not myofibril length-dependent but there is mutual alignment of fibers (*L* = OFF, *τ* = ON); and 3) there is neither fiber force-length dependence nor any mutual alignment of fibers (*L* = OFF, *τ* = OFF). In simulations where the nascent myofibrils have fiber force-length dependence, fibers will predominantly organize along the major diagonal (Fig. 5A) as shown experimentally (Fig. 2B), however, when there is no fiber force-length dependence, fiber bundles follow both the long and short diagonals (Fig. 5B).

We compared the mean degree of parallel coupling as a function of time for all conditions (Fig. 5C). This analysis reveals that the force-fiber length dependence is an essential contributor to the emergence of an organized equilibrium in the myofibrillar network. In these simulations, the absence of the force-length dependence potentiated a less organized nascent myofibril network, whereas mutual alignment of nascent myofibrils enhanced parallel coupling. Eliminating the mutual alignment alone (grey dot-dashed line), produces a minor effect in the stair cell as shown in the inset of Fig. 5C, however, previous reports suggest that the effect of mutual fiber alignment is seen at longer time scales [1], [21], [22], [23].

We asked how mutual fiber alignment would effect myofibrillar organization in the circular cell, whose homogeneous boundary curvature requires an internal, random symmetry break to achieve equilibrium. By eliminating the ability of fibers to cooperatively align in circular cells (grey-empty circle line Fig. 5C), we show that the increase in parallel fiber coupling is solely depended on the ability of the nascent myofibrils to mutually align. The importance of mutual alignment is illustrated by contrasting the steady state fiber organization in circle cell with mutual alignment (Fig. 5D) and no mutual alignment (Fig. 5E). In the case of no mutual fiber alignment the fibers in the circular cell remain randomly organized, which is contradicted by experimental results (Fig. 3L and Fig. 4N). In summary, our data suggests that the fiber length-force dependence is necessary to reproduce myofibrillogenesis in all cell shapes, while the importance of mutual fiber alignment effect increases in cells with homogenous boundary conditions.

## Discussion

Muscle morphogenesis is a hierarchal, self-organizing process spanning from nanometer scale conformational changes in proteins to bundled fibers sometimes a meter in length. We reasoned that boundary constraints are a physical signal that is conserved over all of these length scales and spatially organizes this broad range of coupled structures. Based on previous experimental evidence [17], [26], [27], [28], we hypothesized that geometric cues in the extracellular space help organize the assembly of the contractile apparatus in the cytoplasm and developed computational and experimental models to recapitulate these events.

We report that distinct cytoskeletal architectures arise from two temporally-ordered, organizational processes: the cooperative interaction between premyofibrils and focal adhesions, as well as the mutual alignment and parallel bundling of nascent myofibrils. Our model assumes that the assembly of FAs and the parallel bundling of actin based fibers is coupled by a positive feedback loop and that the growing force on the FA potentiates its structural reinforcement, as suggested by previous experimental work [7], [8], [9]. By modeling the amount of bound and unbound integrin and by marking the maturation of the premyofibril to a nascent myofibril simply by increased contractility, we are able to predict the organization of the contractile apparatus in cardiac myocytes cultured on engineered substrates in a computationally efficient manner. To achieve this efficiency, we ignore the details of the molecular constituents of the assembly of myofibrils [2], [3], [4]. However, we were able to account for all the dominant factors in a course grained manner as indicated by the match between all our models and experiments. By experimenting with our assumptions *in silico* and comparing them to data from *in vitro* experiments, our results suggest that the force that the myofibrillar bundle exerts on the substrate is fiber length-dependant [10], [11], [12] and that the adjacent myofibrils exert “torque” on one another to facilitate coupling [24], are necessary to describe how these myocytes build and organize their internal cytoskeleton relative to extracellular cues. Our computationally efficient model recapitulates the elegant protein choreography of the sarcomere assembly, where an ensemble of proteins assembles repetitively along the length of the actin fiber template.

Several models of cell cytoskeleton assembly and mechanics have been reported and it is worthwhile to compare and contrast the efforts [14], [15], [29]. Our model is similar to the model by Novak and colleagues [16] in that we have used reaction kinetics to simulate the dynamic self-assembly and – organization of the cytoskeleton. These approaches differ from that of Deshpande, et al [14], [15], [16] who report a solid mechanics model and Pazek and colleagues [14], [15], [29] who use a mechanochemical model. All four of these models simulate the bound and free states of integrins in some form and also model the increasing stabilization, or maturation, of focal adhesions with increases in exerted force. The Despande and Pazek models offer detailed mechanical analysis of the cell-substrate interface, whereas our model, like the Novak model, does not. While the Pazek, et al., model does not recapitulate stress fibers, our model, like the Novak and Deshpande models, does. Our model accounts for the specialized case of the maturing striated muscle cell by mimicking the transition of a premyofibril to the nascent myofibril, modeled by an increased ability to generate tension. The Hammer and Novak models omit the fiber length-force assumption that is critical to our model's ability to recapitulate our experimental data. Similarly, the Desphande and Novak models explicitly do not account for mutual alignment of fibers, whereas ours does. Our model, like the Desphande et al. and Pazek models, calculates the load exerted on the substrate by the contracting cell, where the Desphande and Pazek models offer detailed descriptions of the solid mechanics at this interface. Both our model and that by Novak et al., are similar to larger scale models of myofibril adaptation in the left ventricle [30], in the assumption that there is a network of fibers where all integrins are connected to all other integrins. Each model, including the one reported herein, varies in approach and further work is required to test all of these models against experimental data as we have attempted.

We were able to reproduce the results shown by Novak et al., [16], who predicted that with no fiber tension-length dependence, and homogeneous boundary conditions the FAs would aggregate to the perimeter. However, as our *in vitro* work shows even with a homogeneous boundary condition, i.e. the circular cell, there occurs a symmetry break, therefore it is necessary to introduce fiber tension-length dependance and mutual alignment of fibers for *in silico* experiments. We can also utilize the model to explore the effect of cell boundary curvature, cell aspect ratios and combinations of multiple cells on the myofibril distribution, as well as the relative importance of mutual fiber alignment in three dimensions. Additionally, it will be possible to integrate our model with adhesion dynamics models using the same methods as Paszek et al., to explore integrin clustering with contractile cells on substrates with different material properties [29]. This combination of a mechanical model with our myofibrillogenesis model could also allow for simulations of the rearrangement of the extracellular matrix by contractile cells.

In summary, our study suggests that hierarchal organization of muscle requires localized cues that guide myofibrillogenesis. Specifically, a local symmetry break is required to potentiate the assembly and organization of FA and actin complexes that are the template for myofibrillar organization. Such cytoskeletal symmetry-breaking has also been widely observed in other important biological behaviors such as cellular migration [11], cellular division [31], and formation of tissue sheets [32]. The symmetry-breaking can arise from a static, external cue, such as a geometric feature in the boundary conditions imposed on the cell, or from a dynamic internal cue, such as a local overlapping of long fibers. The multiple time scales of these interacting events suggest a hierarchy of post-translational, self-organizational processes that are required for coupling cellular form and function.

## Materials and Methods

### Mathematical Description of the Model

#### Model formulation

The model is based on the principles of reaction kinetics. This allows us to track densities (or concentrations) instead of individual molecular constructs. We assume that the focal adhesions are formed by the binding of integrins and that the integrins can exist in a free, 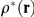, or bound, 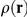, form. The bound integrins are connected to pre-, or nascent, myofibrils via an adsorption process (Eq. (5) & (6)). The myofibrils are force bearing fibers and are approximated by a network which connects every bound integrin to every other bound integrin in the cell [16]. We model two types of myofibrils: pre-myofibrils and nascent myofibrils. Premyofibrils mature into a more stable nascent myofibril which can produce more force [1], [13]. The integrins are represented by three fields: unbound integrins, 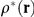 (Eq. (1)), bound integrins connected to pre-myofibrils, 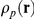 (Eq. (2)), and bound integrins connected to nascent myofibrils, 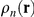 (Eq. (3)). The total number of integrins is held constant throughout the simulation. Bound integrins form FAs and the total density of bound integrins is defined as 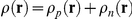. In the unbound state, the integrins diffuse through the 2D cell. Diffusion is assumed to be faster than all other processes in the cell, and therefore it is approximated as instantaneous.

The higher the force exerted on a FA, the more stable it is, i.e. at that point in space the rate of converting unbound integrin to bound integrin is increased [7], [8], [9]. Our hypothesis is that the force produced by each fiber is larger if the fiber is longer, however the model includes the flexibility to test this hypothesis by making the force independent of fiber length, i.e., changing the value of 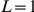 to 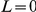 in Eq. (4). The increase in force due to an increase in the number of fibers is bound by the equilibrium of the adsorption process, attenuated by 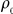 (Eq. (5) & (6)). We introduce a biasing potential field, 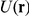 (Eq. (7)), acting on the free integrins, the net effect of which is to cluster focal adhesions together if each has fibers leading to the same distant point. This property can be turned off by setting parameter 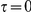 in Eq. (1), or, the property can be adjusted by varying the proximity of the effect, changing the value of 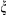. The net force on the integrins is translated to the substrate, and the traction stress vector on the substrate is therefore given by 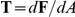 [33]. The model is then expressed as a set of equations, two of which are ODEs, where all variables are defined in Table 1:
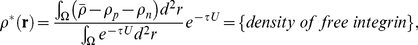
(1)

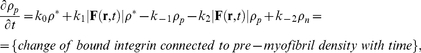
(2)

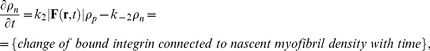
(3)

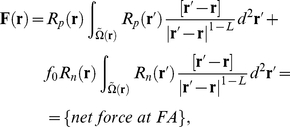
(4)

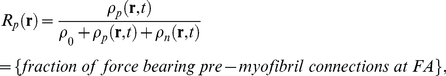
(5)

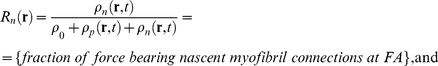
(6)

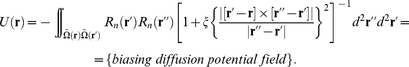
(7)For convex cells integration in Eq. (4) and (7) are performed over the whole cell cultured on an ECM island, i.e. 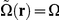. For concave cells the integration is performed only for pairs of points that are connected by fibers that are entirely contained within the ECM island. We can formally represent this concept by defining a 2D space of pairs for each 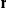:
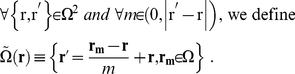
(8)The system of model equations (Eq. (1)–(7)) is discretized and solved using MatLab (Fig. 1H and S3). The details on discretizing the equations and the schematic representation of the MatLab code can be found in the supplemental information (Text S1 and Fig. S3).

**Table 1:** Model variables.

Variable	Definition
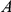	Total cell area, set to unity
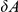	Unit area
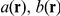	Fourier coefficients of 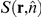
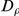	Membrane diffusion coefficient of unbound integrin
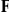	Net force exerted on the bound integrin at 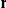
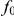	Ratio of contribution to net force of nascent myofibrils to pre-myofibrils
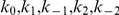	Rate constants for integrins binding to and unbinding from pre- and nascent myofibrils
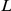	Force is fiber length dependent ( 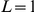 ), or independent ( 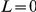 )
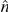	Unit vector indicating the direction of a fiber
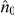	Director of the fibers at point 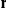
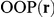	Orientational order parameter of the fibers at 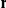
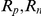	Fraction of bound integrin connected to each type of fiber
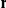	Vector field defining each point in the 2D geometry
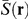	Density of fibers at point 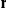
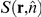	Fraction of fibers in direction 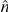 about 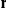
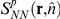 , 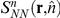 , 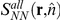	Length of fibers (pre-myofibrils, nascent myofibrils, and all, total, fibers respectively) passing through a unit area 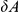 in the direction 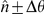 (Not normalized to the total length)
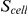	Length of all the fibers inside 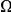 , i.e. inside the cell
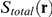	Total length of fibers crossing unit area 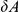 , per unit area
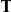	Traction stress at 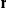
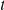 , 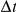 , 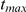	All time-scales, computational time step and the total simulation time
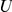	Biasing potential function for mutual fiber alignment
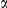	Spatial step along a fiber for integration
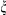	Parameter that sets the size of the potential attraction well (inversely proportional to the affected area)
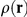	Total density of bound integrin
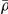	Total average integrin density (constant throughout the simulation)
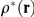	Density of unbound integrin
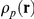	Density of bound integrin connected to pre-myofibrils
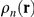	Density of bound integrin connected to nascent myofibrils
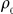	Constant that attenuates the rate of saturation of integrin-fiber connections
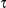	Ratio of the biased diffusion coefficient to the diffusion coefficient
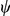	Degree of parallel coupling
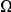	2D defined geometry of the cell in the 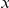 - 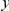 plane
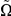	2D defined set for each 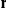 , such that the line segment 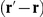 never crosses the empty spaces in the concave cells (for convex cells 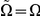 )
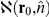	Number of fibers passing through a unit area 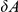 in the direction 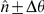

Glossary of parameters and functions in the mathematical model. All vectors are in bold.

#### Model output: Fibril distribution

To calculate the fiber distribution, we use the above assumption that the fibers are approximated by the network connecting all the integrins to each other. To continue to operate with concentration fields instead of individual integrins we calculate the total length of fiber passing through each small area in a specified direction:

pre-myofibril network,
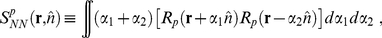
(9)nascent myofibril network,
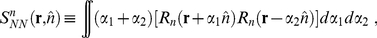
(10)and the total myofibrillar network
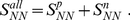
(11)


The rest of the equations describing our method for calculating the properties of the fiber network are the same for all three types of fibers (pre-myofibril, nascent myofibril and overall networks). Therefore, for brevity, we present them only once and a schematic representation of these values can be found in the supplemental information (Fig. S4). The fiber density, 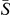, at any point in the cell island is given by the length of fiber passing through the small area around the point of interest, 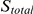, normalized by the total length of fibers in the cell, 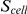:
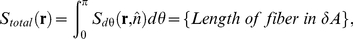
(12)

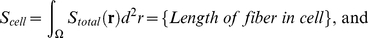
(13)

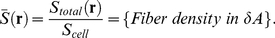
(14)Likewise, the density distribution of fibers in a small area around a given point going in a given direction is calculated by dividing the length of fiber in that direction by the total length of fiber in the small area around that point:
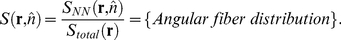
(15)In this model we assume that the network of fibers can be estimated by considering that all integrins are connected to all other integrins. In such a formulation, the fibers can be approximated as straight rods at any given lattice point. The OOP characterizes the degree of order of a distribution of rods, and is zero for perfectly isotropic systems and one for completely aligned rods. We calculate the OOP and the director of the fiber distribution for each point in the cell [34]. The director is the main orientation of a distribution of rods. We perform these calculations by using the coefficients of the Fourier series of the fiber density distribution, 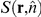:
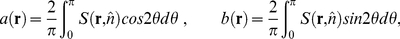
(16)

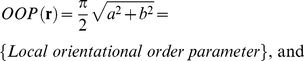
(17)

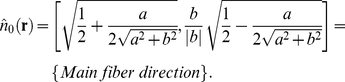
(18)


In drawing the fiber distributions, we assume that at any given point the fibers approximately follow the main direction of the fiber distribution in the small area around that point (Eq. (18)). We define the degree of local parallel coupling as the product of the normalized nascent myofibril density and their degree of order:
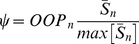
(19)


#### Model parameters

The parameters were fit using a stair-shaped myocyte (Fig. 2), the detailed description can be found in the supplemental information (Text S1). The parameter fit was validated using three other shapes: square cell, triangular cell, and a circular cell. Additionally, the hypotheses were tested by adjusting the appropriate parameters on the circular and stair shape cells. Parameter sensitivity studies are described in the supplemental information (Text S1).

The model parameters were fitted using coarse grained variables. The following parameters specify units in the simulations and the computational time step: 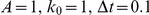. The total computational time for all shapes except the circle was 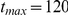, while the circle needed a longer time to achieve equilibrium with 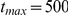. Prior studies suggest that FA formation takes place on a time scale of seconds, followed by the assembly rate of the premyofibril (∼minutes) and the realignment of the nascent myofibril (10–20 hours) [1], [21], [22], [23]. By construction, the rate constants in Eq. (2) will be dictated by the time formation of the premyofibrils, while the rate constants in Eq. (3) will be dictated by the formation time of the nascent myofibrils. The rest of the constants were fitted by matching the fiber distribution in the stair shape cell (Fig. 2): 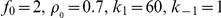, 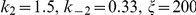. The following parameters were varied to test the hypotheses: 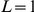 or 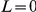, 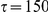 or 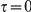.

### Detailed Explanation of Equations

#### Equation (1)

This equation was originally written as,
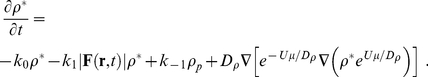
(20)However, it was simplified using the assumption that the diffusion of unbound integrin is much faster than the formation of bound integrin and formation of pre-myofibrils and nascent myofibrils.

To arrive at Eq. (1), we assume all the terms are small compared to the diffusion term and that the mass is conserved. The mass conservation can be written as the following for each time step, where the total amount of integrin does not change:
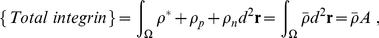
(21)

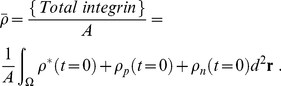
(22)Together we arrive at:
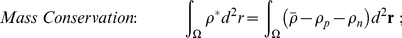
(23)

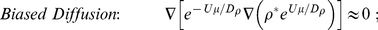
(24)

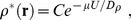
(25)

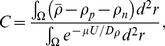
(26)where, 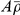 is the total amount of all types of integrins in the cell, 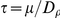 is the ratio of the bias diffusion and the free diffusion constants.

#### Equations (2)–(3) and equation (20)

The first term in Eq. (2) and in Eq. (20): at point 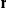 the rate of conversion of free integrins to premyofibril-connected bound integrins increases as the density of free integrins increases. The second term in Eq. (2) and in Eq. (20): a larger force promotes the conversion of unbound integrin to bound integrin connected to pre-myofibrils, or in other words makes the bound integrin more stable. The third term in Eq. (2) and Eq. (20): the more bound integrin there is at point 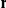 the higher the rate of its conversion to unbound integrin.

The fourth term in Eq. (20) is the diffusion of the unbound integrin. In this term 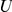 is the biasing potential field that forces a distribution of free integrins that biases the fibers towards co-aligning with each other. The fourth term in Eq. (2) and first term in Eq. (3): the more force on a focal adhesion at 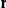 the higher the rate of conversion from the pre-myofibrils to nascent myofibrils, and the bound integrins change from being connected to pre-myofibril to be connected to nascent myofibrils. The fifth term in Eq. (2) and second term in Eq. (3): the more nascent myofibrils there are the higher the rate of conversion back to pre-myofibrils, i.e. the bound integrins change from being connected to nascent myofibril to be connected to pre-myofibrils.

#### Equations (4)–(6)

In Eq. (4) the force was normalized such that 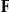 has the same units as 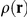. Here we assume that the bound integrins that contribute to the force are the ones that are connected to the myofibrils. The fraction of bound integrins connected to the pre-myofibrils and nascent myofibrils is given by the Langmuir isotherms in Eq. (5) & (6), respectively.

The first integral term in Eq. (4) is the force contributed by the pre-myofibrils: 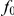 is the relative strength of the pre-myofibril and nascent myofibril. The force at 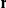 is calculated by a vector sum (integral) of all the contributions from all other integrins. The force between 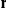 and 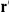 is given by the number of connections between those two points scaled by the distance between the points. The “number” of connections is basically 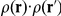. However, there is a limit of how many fibers can connect to any point 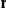, this bounded quantity is given by the function 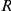.

The second integral term of Eq. (4) is the same as the first, but it calculates the force contribution from the nascent myofibrils. In Eq. (5) and Eq. (6), 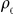 is the inverse of the equilibrium constant of the “adsorption” process of bound integrins connecting to the fibers. The numerator of Eq. (5) and Eq. (6) is the pre-myofibril bound integrin and nascent myofibril bound integrin, respectively. Also, note that 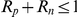. The speed at which the saturation value is reached depends on the 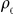. At its limit 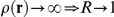. Note that the density of bound integrins connected to the fibers would be given by 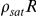, where 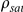 is a constant specifying the total available connections between the bound integrin and fiber in the unit area. This constant is not present in the equations as it is rolled into dimensionalization of 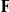.

#### Equation (7)

The term in the figure parenthesis in Eq. (7) is simply the distance between point 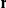 and the line 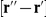. The biasing potential on point 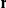 is stronger if the fiber is “thicker,” thus the amount of binding at each end-point is taken into account. The form of the biasing potential is such, that it is low for 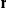 close to fiber 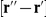, and zero far away. The area around each fiber where the potential is not zero is inversely proportional to 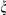. The total biasing potential on point 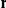 is the sum of contribution by each nascent myofibril.

#### Equation (9)–(15)

While numerically it is easiest to operate with density fields, it is easier to understand this equation by first writing the expression for the number of fibers passing through an area 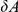 about a point 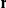, in the direction 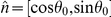. In the following equation the area on one side of 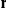 in the direction 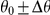 and up to the boundary of the cell is 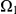 and in the other direction 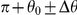 is 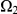. The number of integrins at each point, 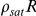 is taken into account by assuming the number of fibers passing between the two points is the product of the number of fibers at each point. To get to Eq. (9)–(10), we must account for the length of the fiber passing through 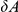,
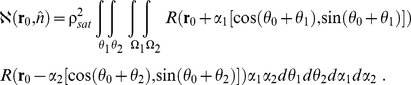
(27)The rest of equations are a manipulation with the length of fibers in the space 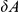 and the whole cell Ω.

#### Equation (16)–(18)

To find the fiber directions and the degree of alignment, we consider the Fourier series of the fiber distribution 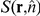:
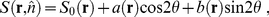
(28)where, *a*, *b* are constant at each 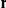. The first term of the series is simply the average length of the fiber in the unit area, 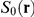, and is given simply by:
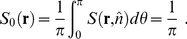
(29)The next two terms contain information about the main direction of the fibers and the degree of orientation and given in Eq. (16). The director is the direction, such that rotating to that frame of reference leaves only the cosine term of the Fourier series. The orientational order parameter (OOP) is the Fourier coefficient in such a frame of reference:
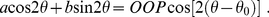
(30)We can use a trigonometrical identity to show this and to solve for the coefficients. We then normalize the OOP to be between zero (isotropic) to one (perfect alignment):
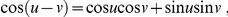


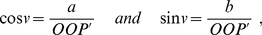
(31)

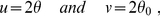
(32)

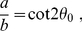
(33)

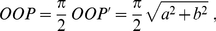
(34)

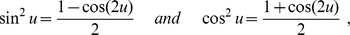


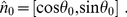
(35)Note that physically 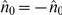, and therefore the outer square root in Eq. (18) can be any sign as long as we are consistent.

### Cardiac Myocyte Culture

All experiments were conducted in accordance with the guidelines of the Institutional Animal Care and Use Committee of Harvard University. Trypsinized ventricular tissue isolated from 2-day old neonatal Sprague Dawley rats (Charles River Laboratories, Wilmington, MA) was serially dissociated into single cells by treating the ventricular tissue 4 times with a 0.1% solution of collagenase type II (Worthington Biochemical, Lakewood, NJ) for 2 minutes at 37°C. The myocyte fraction was purified and pre-plating the cells twice for 45 minutes each time. Purified myocytes were plated onto micropatterned substrates prepared as described below at a density of 100,000 cells per coverslip and kept in culture at 37°C with a 5% CO_2_ atmosphere. The culture medium was M199 (Invitrogen, Carlsbad, CA) base supplemented with 10% heat-inactivated Fetal Bovine Serum, 10 mM HEPES, 20 mM glucose, 2mM L-glutamine, 1.5µM vitamin B-12, and 50 U/ml penicillin. The medium was changed 24 hours after plating to remove unattached and dead cells and every 48 hours afterwards. After 72 hours in culture, most cardiac myocytes beat spontaneously and were used either for immunostaining or traction force measurements.

### Micropatterning Substrates

Micropatterned substrates containing square, triangular, or circular adhesive islands were prepared for immunostaining and traction force microscopy, as follows. For immunostaining, the substrates were micropatterned using a microcontact printing procedure similar to that described by Tan *et al.* [35]. Micropatterned substrates for traction force experiments were created by adapting the published techniques [10], [36]. Briefly, a thin layer of 10% by weight poly-N-iso- propylacrylamide (PIPAAM) prepared in 1-butanol was spin coated on a silicon wafer (Fig. S1a). A 50∶75 µm layer of photoresist (SU-8, MichroChem Corp, Newton, MA) was spin-coated on top of the PIPAAM (Fig. S1b), UV light treated through a photolithographic mask (Fig. S1c), and developed to obtain a complementary master that contained holes with the same size and shape as the desired adhesive islands (Fig. S1d). The master was immersed in ice water to dissolve the PIPAAM and the photoresist membrane was released from the wafer (Fig. S1e). Polyacrylamide gels (0.1% bis and 5% acrylamide; 90 µm thick) containing 1∶500 volume of carboxylate-modified fluorescence latex beads (0.2 µm Fluospheres, Molecular Probes, Eugene, OR) were fabricated on 25 mm coverslips. The Young's modulus of the gel was estimated to be ∼3 KPa using atomic force microscopy as described previously [37]. The photoresist membrane was placed on the surface of the gel and 1 mM sulfo-SANPAH (sulfosuccinimidyl- 6-4-azido-2-nitrophenylamino-hexanoate; Pierce, Rockford, IL) in 50 mM HEPES was added through the holes in the photoresist membrane. The whole system was then placed under vacuum for 3 minutes to ensure that the sulfo-SANPAH reached the gel surface. The surface of the gel that contacted with the sulfo-SANPAH was photoactivated by UV light exposure (Fig. S1f). After excess sulfo-SANPAH was removed, fibronectin (FN) 100 µg/mL was added to the membrane and the gel was placed under vacuum for another 3 minutes to remove bubbles from the holes (Fig. S1g). The FN was allowed to react with the photoactivated gel for at least 4 hours at 37°C to create FN-coated adhesive islands. Excess FN was washed away with PBS. After removal of the photoresist membrane, the gel was immediately used for cell plating (Fig. S1h).

### Sarcomere Length Measurement

*In vitro* studies show that the maturation of sarcomere can be determined by measuring the distance between two adjacent α-actinin rich spots that are supposed to be the precursors of the Z-band [13]. A 2–2.5 µm spacing between the sarcomeric α-actinin rich spots indicates a matured sarcomere [2]. We used a fast Fourier transform (FFT) to calculate the spacing of sarcomeric 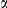-actinin rich spots in Fig. 4A, F, K. An intensity profile of the sarcomeric α-actinin stains was chosen along myofibrils spanning the long axis of the cells. The profile was then detrended, weighted with a Hamming window and transformed into the spatial frequency domain by FFT. The spatial frequency at peak power of the first-order harmonic in the spatial frequency domain was identified and converted into the spatial domain to yield the sarcomere length. The results reveal that the sarcomere lengths are 2.4±0.1 µm, 2.2±0.1 µm, and 2.4±0.2 µm for the cell in Fig. 4A, F, K, respectively, indicating that they are mature sarcomeres.

### Traction Force Microscopy Data Measurement and Analysis

Coverslips containing the beating myocytes were removed from the incubator, mounted onto a custom-made microscope stage containing a bath chamber, and continuously perfused with 37°C normal Tyrode's solution (1.192 g of HEPES, 0.901 g of glucose, 0.265 g of CaCl_2_, 0.203 g of MgCl_2_, 0.403 g of KCl, 7.889 g of NaCl and 0.040 g of NaH_2_PO_4_ per liter of deionized water, reagents from Sigma, St. Louis, MO). Fluorescence images of gels containing fluorescent beads immediately beneath the contracting myocytes were taken at 28.1 Hz. The duration of image acquisition was long enough to include at least two complete cycles of contraction-relaxation of individual myocytes. Consecutive images were paired and the prior image was used as a reference to measure the change of the position of the fluorescence beads using the algorithm described previously [38]. This yielded the discretized displacement field between two consecutive frames. The calculated displacements were summed up for a whole systolic cycle to determine the overall 2D displacement field. The systolic traction field was calculated from the displacement field by adapting the algorithm previously developed [39], [40]. This algorithm solved the inverse of the Boussinesq solution from the displacement field on the surface of an elastic halfspace to obtain the traction field when the mechanical properties of the gel are known. The Poisson ratio of the gel was assumed to be close to 0.5 [10]. The interior of the cell was subdivided into 4×4 µm^2^ squares to approximate the discretized localization of contractile forces. The ability of a particular solved traction field to explain the observed displacements was estimated with 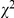 statistics. In addition to a zero-order Tikhonov regularization, a constraint that the forces should not become exceedingly large was used to minimize and stabilize the solution [40]. The *L*-curve criterion, as previously described [40], was used to determine the optimal balance between the data agreement and the regularization.

### Immunofluorescent Staining and Imaging

Cardiac myocytes stained for actin (Alexa 488 Phalloidin, Molecular Probes), vinculin (clone hVIN-1, Sigma), and sarcomeric α-actinin (clone EA-53, Sigma) were fixed in 4% PFA with 0.01% Triton X-100 in PBS buffer at 37°C for 15 minutes and equilibrated to room temperature during incubation. Secondary staining was performed using tetramethylrhodamine- conjugated goat anti-mouse IgG (Alexa Fluor 594, Molecular Probes), and nuclei were visualized by staining with 4′,6′-diamidino-2- phenylindole hydrochloride (DAPI, Molecular Probes). All fluorescence and traction force microscopy was conducted with a Leica DMI 6000B microscope, using a 63× plan-apochromat objective. For traction force experiments, images were collected with a Cascade 512b enhanced CCD camera, while immunofluorescence images were collected with a CoolSnap HQ CCD camera (both from Roper Scientific, Tucson, AZ) controlled by IPLab Spectrum (BD Biosciences/Scanalytics, Rockville, MD).

## Supporting Information

Figure S1 Schematic representation of micropatterning FN on polyacrylamide gel. After a thin layer of PIPAAM was spin-coated on a silicon wafer (a), SU-8 photoresist was spin-coated on top of the PIPAAM (b), treated with UV light through a photolithographic mask (c), and developed to obtain a complementary master (d). The master was immersed in ice water to release the photoresist membrane (e). The photoresist membrane was placed on the surface of polyacrylamide gels and sulfo-SANPAH was added to the gel surface, photoactivated by UV light (f). FN solution was then added to react with the photoactivated gel (g). After removal of the photoresist membrane, the gel was immediately used for cell plating (h).
(5.99 MB TIF)

Figure S2 Comparison of different initial conditions in the stair shape cell. *First column*: Initial condition name. *Second column*:The fiber map at the first time step at which fibers exist (time listed next to each frame). *Third column*: Steady state fiber distribution with a the grey scale showing the degree of parallel coupling. The steady states are the same for each initial condition. *Fourth column*: Map of initial density of free integrins. *Fifth column*: Map of initial density of bound integrins.
(1.00 MB TIF)

Figure S3 Schematic of model implementation algorithm. This schematics shows how each equation was implemented inside the MatLab code.
(0.18 MB TIF)

Figure S4 Schematic showing fiber density and distribution definitions. A: All the fibers in the cell are represented as green lines. The total length of all fibers in the cell is labeled as S_cell_. We consider a point **r**, with an area δA that is vanishingly small for continues systems. For discrete systems δA is the area of cell divided by the number of points in the lattice. B: The total length of fibers inside the area associated with point **r** is the total length of red line segments. C: The length of fibers passing through a small area around point r in the direction of *n* is the total length of the blue lines.
(0.30 MB TIF)

Text S1 The supporting text includes details on implementation of the model in code, and a discussion of parameter sensitivity.
(0.04 MB PDF)

Video S1 Myofibril organization in an equilateral triangular muscle cell. The positive feedback loop between assembly of contractile elements and FA maturation permits continued lengthening of the contractile fibers, with the longest dimension of the cell acting as the only limiting factor to fiber elongation. This is demonstrated in Video S1, where nascent myofibril bundling and orientation were initially random (*t* = 0); as time elapsed, they reoriented themselves and were stabilized along the cellular peripheries at *t* = 120au. The color scale and lines represent the degree of parallel coupling and local orientation of myofibrils, respectively; color values are in arbitrary units.
(1.14 MB AVI)

Video S2 FA organization in an equilateral triangular muscle cell. The color scale represents FA density and color values are in arbitrary units. Initially, FAs were randomly distributed, then were redistributed to the cellular peripheries and accumulated at the cellular corners at steady state. This is because growth of FAs depends on the traction field, defined as the sum of all contractile element vectors connecting to a FA. Thus, FA density is expected to be larger at cellular peripheries with higher curvatures, where the overall alignment of contractile element vectors is also larger, leading to a higher net traction.
(0.62 MB AVI)

Video S3 Myofibril organization in a square muscle cell. Nascent myofibrils were not aligned initially, but at equilibrium, they realigned with enhanced parallel bundling occurring along the diagonals and edges of the cell. Definitions of the color scale and lines are the same as Video S1.
(1.52 MB AVI)

Video S4 FA organization in a square muscle cell. The initial homogenously distributed FAs quickly redistributed to the cellular peripheries and were stabilized at the cellular corners at steady state. Definition of the color scale is the same as Video S2.
(0.56 MB AVI)

Video S5 Myofibril organization in a circular muscle cell. Parallel bundled nascent myofibrils first occurred at the center of the cell, then realigned adjacent fibers, and finally extended across the diameter of the cell to define a principal axis of contraction at *t* = 500au. Note that definitions of the color scale and lines are the same as Video S1.
(1.57 MB AVI)

Video S6 FA organization in a circular muscle cell. FAs in a circular cell first redistributed to the peripheries of the cell. Transient multi-pole patterns of FA then developed in accordance with the reorganized myofibril network as shown in Video S5. These poles were seen to redistribute, merge, and finally converged to a bipolarized pattern with two opposing bands of FA along the cellular peripheries. Definition of the color scale is the same as Video S2.
(2.54 MB AVI)

Video S7 DIC images of a beating triangular myocyte. Images were acquired at 21.7 frames per second and contain a full cycle of contraction and relaxation. Time is labeled at the top left corner. During contraction, the cellular body was shortened toward the center of the cell, with obvious deformation of the nucleus. The cell was still an equilateral triangle at the full contraction, with about 24% shortening along the cellular edges.
(0.33 MB MPG)

Video S8 DIC images of a beating square myocyte. Images were acquired at the same frame rate as Video S7 and contain a full cycle of contraction and relaxation. At full contraction, the cell kept a square shape, with about 18% shortening along the diagonal.
(0.12 MB MPG)

Video S9 Displacements maps of a beating triangular myocyte. The white arrows depict the frame to frame displacements of the fluorescent beads embedded in the gels. The displacements of the beads were not traced individually. Instead, the displacement map was discretized as suggested by Butler *et al.* 5. The color scale represents the magnitude of the displacement vectors. For consistency, the ranges of the color scale are the same for Videos S9, S10, and S12. During systole, the displacements are relatively larger at the cellular corners. Images contain a full contraction cycle.
(0.49 MB MPG)

Video S10 Displacements maps of a beating square myocyte. As seen in the triangular cell (Video S9), larger displacements occurred at the cellular corners during systole. Definitions of the white arrows and color scale are the same as Video S9 and the images represent a full contraction cycle.
(0.44 MB MPG)

Video S11 DIC images of a beating circular myocyte. Images were acquired at the same frame rate as Video S11 and contain a full cycle of systole and diastole. The cellular body was shortened concentrically during systole, with about 8% shortening along the vertical axis and 13% along the horizontal axis.
(0.22 MB MPG)

Video S12 Displacements maps of a beating circular myocyte. Definitions of the white arrows and color scale are the same as Video S9. The displacements at the opposing peripheries on the horizontal axis were relatively larger, and thus defined the principal axis of contraction.
(0.51 MB MPG)

## References

[1] Dabiri GA, Turnacioglu KK, Sanger JM, Sanger JW (1997). Myofibrillogenesis visualized in living embryonic cardiomyocytes.. Proc Natl Acad Sci USA.

[2] Sanger JW, Kang SM, Siebrands CC, Freeman N, Du AP (2005). How to build a myofibril.. J Muscle Res Cell M.

[3] Moerman D, Williams B (2006). Sarcomere assembly in C. elegans muscle.. http://www.wormbook.org.

[4] Holtzer H, Hijikata T, Lin ZX, Zhang ZQ, Holtzer S (1997). Independent assembly of 1.6 mu m long bipolar MHC filaments and I-Z-I bodies.. Cell Struct Funct.

[5] Russell B, Motlagh D, Ashley WW (2000). Form follows function: how muscle shape is regulated by work.. J Appl Physiol.

[6] Schaper J, Froede R, Sthein, Buck A, Hashizume H (1991). Impairment of the myocardial ultrastructure and changes of the cytoskeleton in dilated cardiomyopathy.. Circulation.

[7] Engler AJ, Griffin MA, Sen S, Bonnetnann CG, Sweeney HL (2004). Myotubes differentiate optimally on substrates with tissue-like stiffness: pathological implications for soft or stiff microenvironments.. J Cell Biol.

[8] Quach NL, Rando TA (2006). Focal adhesion kinase is essential for costamerogenesis in cultured skeletal muscle cells.. Dev Biol.

[9] Ramachandran I, Terry M, Ferrari MB (2003). Skeletal muscle myosin cross-bridge cycling is necessary for myofibrillogenesis.. Cell Motil Cytoskel.

[10] Wang N, Ostuni E, Whitesides GM, Ingber DE (2002). Micropatterning tractional forces in living cells.. Cell Motil Cytoskel.

[11] Parker KK, Brock AL, Brangwynne C, Mannix RJ, Wang N (2002). Directional control of lamellipodia extension by constraining cell shape and orienting cell tractional forces.. Faseb J.

[12] Thery M, Pepin A, Dressaire E, Chen Y, Bornens M (2006). Cell distribution of stress fibres in response to the geometry of the adhesive environment.. Cell Motil Cytoskel.

[13] Rhee D, Sanger JM, Sanger JW (1994). The Premyofibril - Evidence for its role in myofibrillogenesis.. Cell Motil Cytoskel.

[14] Deshpande VS, McMeeking RM, Evans AG (2006). A bio-chemo-mechanical model for cell contractility.. Proc Natl Acad Sci USA.

[15] Deshpande VS, Mrksich M, McMeeking RM, Evans AG (2008). A bio-mechanical model for coupling cell contractility with focal adhesion formation.. J Mech Phys Solids.

[16] Novak IL, Slepchenko BM, Mogilner A, Loew LM (2004). Cooperativity between cell contractility and adhesion.. Phys Rev Lett.

[17] Geisse NA, Sheehy SP, Parker KK (2009). Control of myocyte remodeling in vitro with engineered substrates.. In Vitro Cell Dev-An.

[18] Chrzanowska-Wodnicka M, Burridge K (1996). Rho-stimulated contractility drives the formation of stress fibers and focal adhesions.. J Cell Biol.

[19] Galbraith CG, Yamada KM, Sheetz MP (2002). The relationship between force and focal complex development.. J Cell Biol.

[20] Burridge K, ChrzanowskaWodnicka M (1996). Focal adhesions, contractility, and signaling.. Annu Rev Cell Dev Biol.

[21] Balaban NQ, Schwarz US, Riveline D, Goichberg P, Tzur G (2001). Force and focal adhesion assembly: a close relationship studied using elastic micropatterned substrates.. Nat Cell Biol.

[22] McKenna NM, Wang YL (1986). Possible Translocation of Actin and Alpha-Actinin Along Stress Fibers.. Exp Cell Res.

[23] Hotulainen P, Lappalainen P (2006). Stress fibers are generated by two distinct actin assembly mechanisms in motile cells.. J Cell Biol.

[24] Verkhovsky AB, Svitkina TM, Borisy GG (1995). Myosin-II filament assemblies in the active lamella of fibroblasts - their morphogenesis and role in the formation of actin filament bundles.. J Cell Biol.

[25] Borukhov I, Bruinsma RF, Gelbart WM, Liu AJ (2005). Structural polymorphism of the cytoskeleton: A model of linker-assisted filament aggregation.. Proc Natl Acad Sci USA.

[26] Parker KK, Tan J, Chen CS, Tung L (2008). Myofibrillar architecture in engineered cardiac myocytes.. Circ Res.

[27] Bray MA, Sheehy SP, Parker KK (2008). Sarcomere alignment is regulated by myocyte shape.. Cell Motil Cytoskel.

[28] Bray M-AP, Adams WJ, Geisse NA, Feinberg AW, Sheehy SP (2010). Nuclear morphology and deformation in engineered cardiac myocytes and tissues.. Biomaterials.

[29] Paszek MJ, Boettiger D, Weaver VM, Hammer DA (2009). Integrin clustering is driven by mechanical resistance from the glycocalyx and the substrate.. PLoS Comput Biol.

[30] Kroon W, Delhaas T, Bovendeerd P, Arts T (2009). Computational analysis of the myocardial structure: Adaptation of cardiac myofiber orientations through deformation.. Med Image Anal.

[31] Thery M, Jimenez-Dalmaroni A, Racine V, Bornens M, Julicher F (2007). Experimental and theoretical study of mitotic spindle orientation.. Nature.

[32] Weber KL, Fischer RS, Fowler VM (2007). Tmod3 regulates polarized epithelial cell morphology.. J Cell Sci.

[33] Love AEH (1927). A treatise on the mathematical theory of elasticity.

[34] Umeno A, Ueno S (2003). Quantitative analysis of adherent cell orientation influenced by strong magnetic fields.. IEEE Trans Nanobiosci.

[35] Tan JL, Liu W, Nelson CM, Raghavan S, Chen CS (2004). Simple approach to micropattern cells on common culture substrates by tuning substrate wettability.. Tissue Eng.

[36] Pelham RJ, Wang YL (1997). Cell locomotion and focal adhesions are regulated by substrate flexibility.. Proc Natl Acad Sci USA.

[37] Engler A, Bacakova L, Newman C, Hategan A, Griffin M (2004). Substrate compliance versus ligand density in cell on gel responses.. Biophys J.

[38] Butler JP, Tolic-Norrelykke IM, Fabry B, Fredberg JJ (2002). Traction fields, moments, and strain energy that cells exert on their surroundings.. Am J Physiol-Cell Ph.

[39] Dembo M, Wang YL (1999). Stresses at the cell-to-substrate interface during locomotion of fibroblasts.. Biophys J.

[40] Schwarz US, Balaban NQ, Riveline D, Bershadsky A, Geiger B (2002). Calculation of forces at focal adhesions from elastic substrate data: The effect of localized force and the need for regularization.. Biophys J.

