# Supplementary figures and images for: Self-Organization of Muscle Cell Structure and Function
Source: PLoS Comput Biol. 2011 Feb 24;7(2):e1001088. doi: 10.1371/journal.pcbi.1001088 (PMC3044763; doi:10.1371/journal.pcbi.1001088)

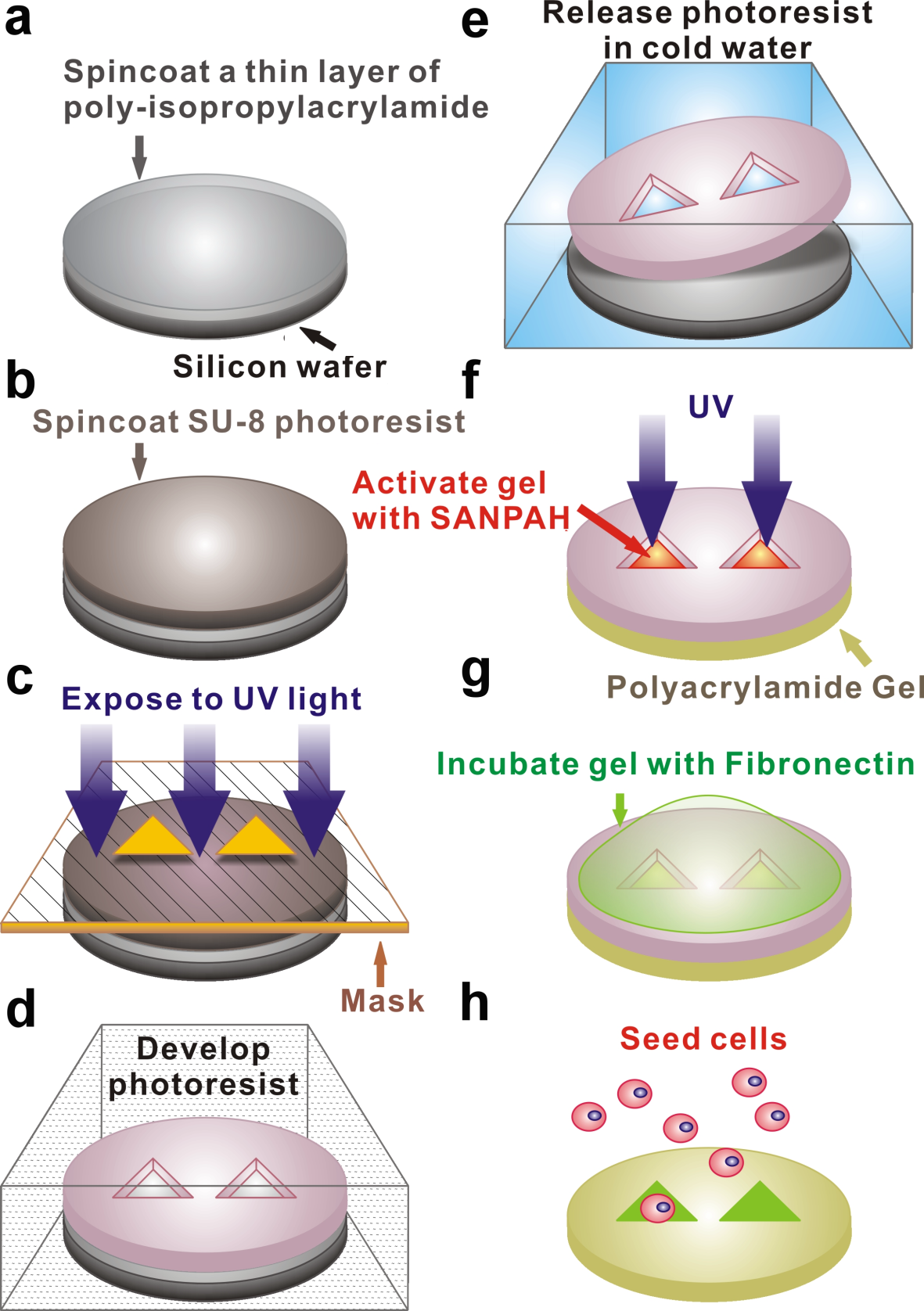

Supplement: Figure S1 — Schematic representation of micropatterning FN on polyacrylamide gel. After a thin layer of PIPAAM was spin-coated on a silicon wafer (a), SU-8 photoresist was spin-coated on top of the PIPAAM (b), treated with UV light through a photolithographic mask (c), and developed to obtain a complementary master (d). The master was immersed in ice water to release the photoresist membrane (e). The photoresist membrane was placed on the surface of polyacrylamide gels and sulfo-SANPAH was added to the gel surface, photoactivated by UV light (f). FN solution was then added to react with the photoactivated gel (g). After removal of the photoresist membrane, the gel was immediately used for cell plating (h). (5.99 MB TIF) [file pcbi.1001088.s001.tif]

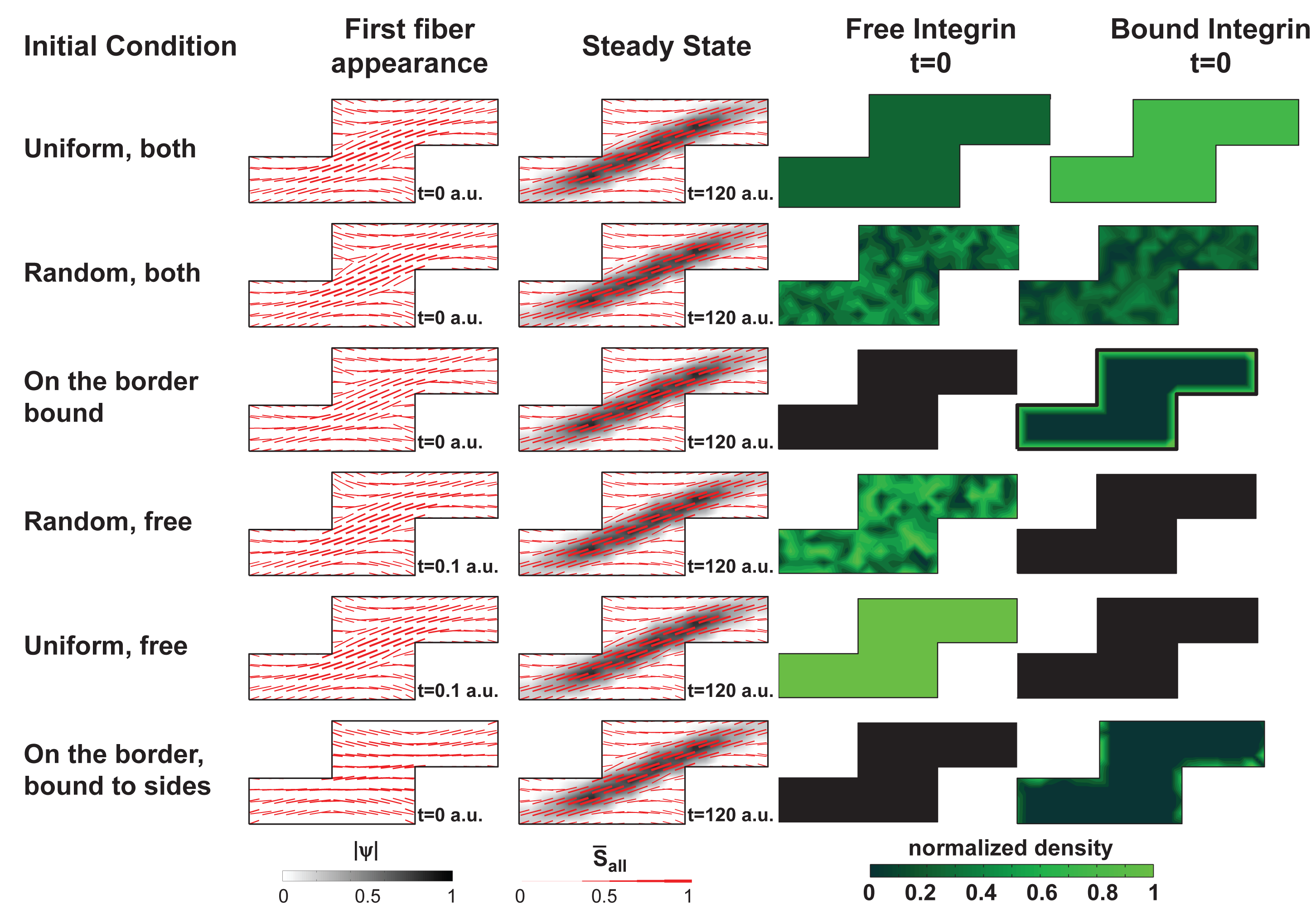

Supplement: Figure S2 — Comparison of different initial conditions in the stair shape cell. First column: Initial condition name. Second column:The fiber map at the first time step at which fibers exist (time listed next to each frame). Third column: Steady state fiber distribution with a the grey scale showing the degree of parallel coupling. The steady states are the same for each initial condition. Fourth column: Map of initial density of free integrins. Fifth column: Map of initial density of bound integrins. (1.00 MB TIF) [file pcbi.1001088.s002.tif]

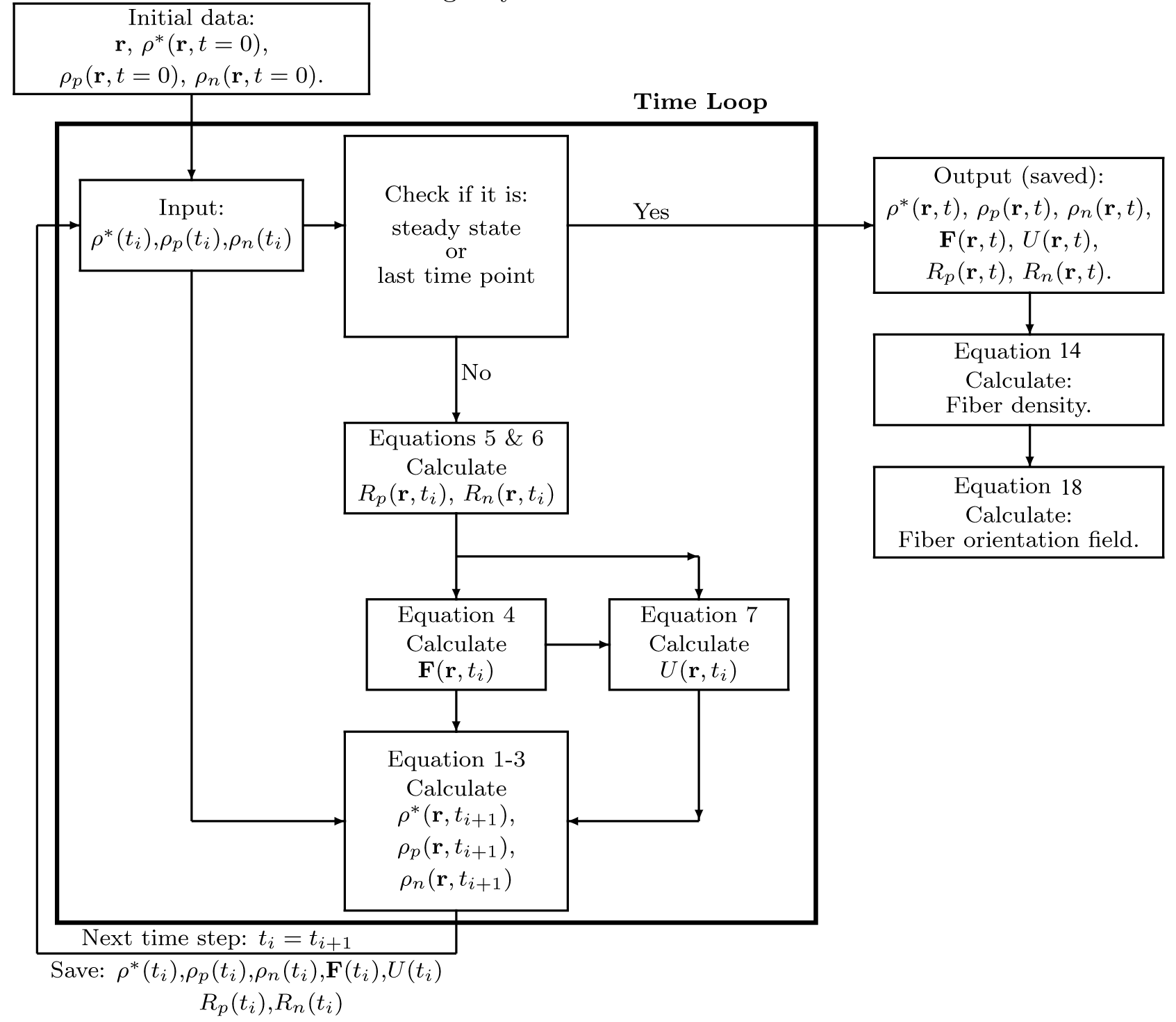

Supplement: Figure S3 — Schematic of model implementation algorithm. This schematics shows how each equation was implemented inside the MatLab code. (0.18 MB TIF) [file pcbi.1001088.s003.tif]

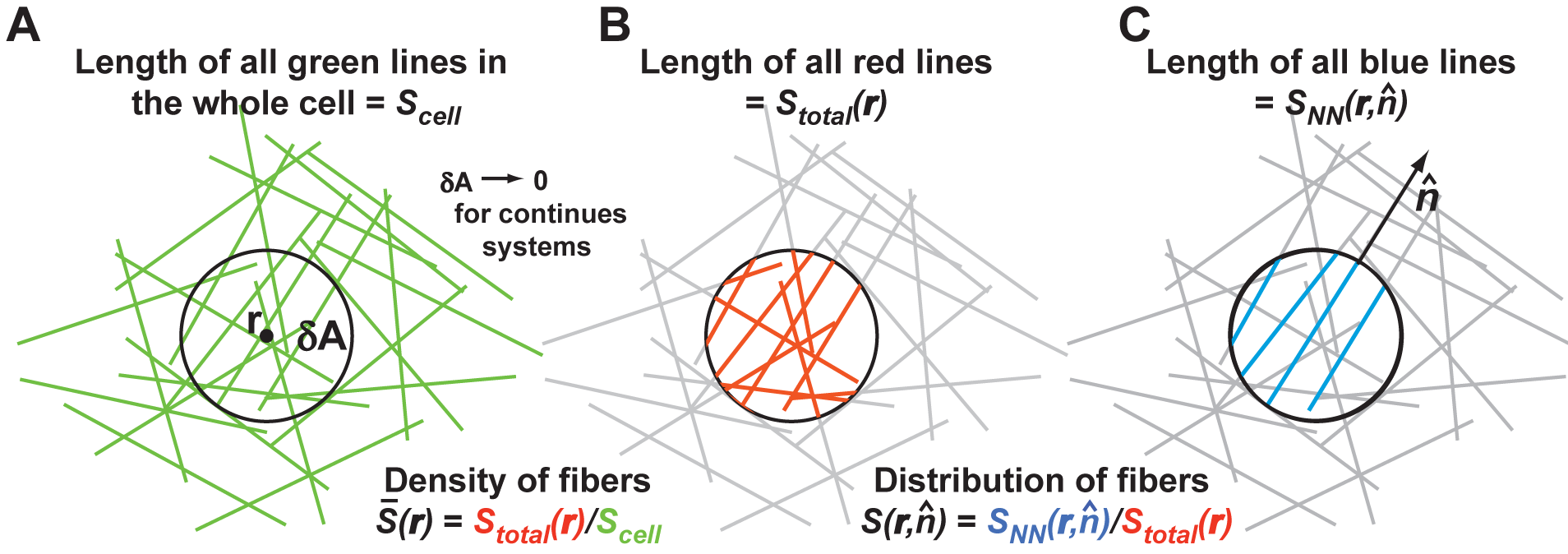

Supplement: Figure S4 — Schematic showing fiber density and distribution definitions. A: All the fibers in the cell are represented as green lines. The total length of all fibers in the cell is labeled as Scell. We consider a point r, with an area δA that is vanishingly small for continues systems. For discrete systems δA is the area of cell divided by the number of points in the lattice. B: The total length of fibers inside the area associated with point r is the total length of red line segments. C: The length of fibers passing through a small area around point r in the direction of n is the total length of the blue lines. (0.30 MB TIF) [file pcbi.1001088.s004.tif]
