# Supplementary material for: Self-Organization of Muscle Cell Structure and Function
Source: PLoS Comput Biol. 2011 Feb 24;7(2):e1001088. doi: 10.1371/journal.pcbi.1001088 (PMC3044763; doi:10.1371/journal.pcbi.1001088)
Supplement: Text S1 — The supporting text includes details on implementation of the model in code, and a discussion of parameter sensitivity. (0.04 MB PDF) [file pcbi.1001088.s005.pdf]

Supporting Text S1:

The supporting text includes details on implementation of the model in code, and a discussion of parameter sensitivity.

Implementation of equations in a discrete setting

Model equations

The equations that contain integrals need to be re-written for the discrete setting to become a sum. The following is the equation for the force re-written from Eq. (4), note that the sum is multiplied by the unit area $\delta A = A/\{Number\ of\ \ points\}$:

$$\mathbf{F}(\mathbf{r}) = R_p(\mathbf{r}) \sum_{\forall \mathbf{r}' \in \tilde{\Omega}(\mathbf{r})} R_p(\mathbf{r}')[\mathbf{r} - \mathbf{r}']\delta A + f_0 R_n(\mathbf{r}) \sum_{\forall \mathbf{r}' \in \tilde{\Omega}(\mathbf{r})} R_n(\mathbf{r}')[\mathbf{r} - \mathbf{r}']\delta A \,. \tag{36}$$

When the length dependence is turned off the above equation becomes:

$$\mathbf{F}(\mathbf{r}) = R_p(\mathbf{r}) \sum_{\forall \mathbf{r}' \in \tilde{\Omega}(\mathbf{r})} R_p(\mathbf{r}') \frac{[\mathbf{r} - \mathbf{r}']}{|\mathbf{r} - \mathbf{r}'|}\delta A + f_0 R_n(\mathbf{r}) \sum_{\forall \mathbf{r}' \in \tilde{\Omega}(\mathbf{r})} R_n(\mathbf{r}') \frac{[\mathbf{r} - \mathbf{r}']}{|\mathbf{r} - \mathbf{r}'|}\delta A \,. \tag{37}$$

Likewise, the biasing potential is numerically calculated as:

$$U(\mathbf{r}) = \sum_{\forall \mathbf{r}' \in \tilde{\Omega}(\mathbf{r})} \sum_{\forall \mathbf{r}'' \in \tilde{\Omega}(\mathbf{r}')} R_n(\mathbf{r}') R_n(\mathbf{r}'') \left[1 + \xi \left\{ \frac{\left| [\mathbf{r} - \mathbf{r}'] \times [\mathbf{r}'' - \mathbf{r}'] \right|}{|\mathbf{r}'' - \mathbf{r}'|} \right\}^2 \right]^{-1} \delta A^2 \,. \tag{38}$$

The traction stress calculation becomes simply $\mathbf{T}(\mathbf{r}) = \mathbf{F}(\mathbf{r})/\delta A$. Note that for the purposes of the numerical code it is easier to rewrite the cross-product term as:

$$\left\{ \frac{\left| [\mathbf{r} - \mathbf{r}'] \times [\mathbf{r}'' - \mathbf{r}'] \right|}{|\mathbf{r}'' - \mathbf{r}'|} \right\}^2 = \left(\frac{1}{|\mathbf{r}'' - \mathbf{r}'|^2} \left| (\mathbf{r} - \mathbf{r}')|\mathbf{r}'' - \mathbf{r}'|^2 - (\mathbf{r}'' - \mathbf{r}')[(\mathbf{r}'' - \mathbf{r}') \cdot (\mathbf{r} - \mathbf{r}')] \right| \right)^2 \,. \tag{39}$$

Fiber distribution

The fiber distribution equations are re-written with the same principle, the use of sums rather than integrals. However, it is important to note that the integral in Eq. (9)-(10), is not over the area of the cell, but along a line passing through $\mathbf{r}$ in the direction of $\hat{n}$. To take such an integral we map the data from the grid $\mathbf{r}$ to a polar grid about each point:

$$S_{NN}(\mathbf{r},\hat{n}) = \Delta\alpha_1\Delta\alpha_2 \sum_{\forall\alpha_1}\sum_{\forall\alpha_2}(\alpha_1+\alpha_2)\left[R(\mathbf{r}+\alpha_1\hat{n})R(\mathbf{r}-\alpha_2\hat{n})\right], \tag{40}$$

$$S_{total}(\mathbf{r}) = \Delta\theta \sum_{\forall\hat{n}} S_{NN}(\mathbf{r},\hat{n}), \tag{41}$$

$$S_{cell} = \delta A \sum_{\forall\mathbf{r}} S_{total}(\mathbf{r}), \tag{42}$$

$$\bar{S}(\mathbf{r}) = \frac{S_{total}(\mathbf{r})}{S_{cell}}, \tag{43}$$

$$S(\mathbf{r},\hat{n}(\theta)) = \frac{S_{d\theta}(\mathbf{r},\hat{n})}{S_{total}(\mathbf{r})}, \tag{44}$$

$$a(\mathbf{r}) = \frac{2\Delta\theta}{\pi}\sum_{\forall\hat{n}} S(\mathbf{r},\hat{n})\cos 2\theta, \qquad b(\mathbf{r}) = \frac{2\Delta\theta}{\pi}\sum_{\forall\hat{n}} S(\mathbf{r},\hat{n})\sin 2\theta d\theta, \tag{45}$$

$$OOP(\mathbf{r}) = \frac{\pi}{2}\sqrt{a^2+b^2}, \tag{46}$$

$$\hat{n}_0(\mathbf{r}) = \left[\sqrt{\frac{1}{2}+\frac{a}{2\sqrt{a^2+b^2}}}, and\ \frac{b}{|b|}\sqrt{\frac{1}{2}-\frac{a}{2\sqrt{a^2+b^2}}}\right]. \tag{47}$$

Model parameter sensitivity

The model parameters were explored for their affect on the results. The following is how each parameter affects the model, however, note that some of the parameters give similar effects by different means:

1. Increasing the rate constant, k_1, increases the rate of conversion of $\rho^* \to \rho_p$, thus shifting the equilibrium towards larger/more concentrated FAs. Decreasing this rate constant has an opposite effect. This also forces the system to come to equilibrium faster.
2. Decreasing the rate constant, k_{-1}, decreases the rate of conversion of $\rho_p \to \rho^*$, thus pushing the equilibrium towards more concentrated FAs. Again, increasing this rate constant will have an opposite effect.
3. Increasing k_2, increases the rate of formation of nascent myofibrils from the pre-myofibrils, or in the renaming of the bound integrins $\rho_p \to \rho_n$.

4. Decreasing k_{-2}, decreases the rate of dissolution of nascent myofibrils, $\rho_n \rightarrow \rho_p$. The relative values of k_2 and k_{-2} dictate the relative amounts of nascent and pre-myofibrils at equilibrium.

5. Increasing f_0 increases the rate of formation of FAs and the rate of conversion of pre-myofibrils to nascent myofibrils with increasing amounts of nascent myofibrils. In our simulations, that means an increase in rates later in the simulation. By making $f_0 < k_{-2}/k_2$ we could actually force the dissociation of nascent myofibrils.

6. Increasing ρ_0 increases the size of the FAs by decreasing the maximal concentration of ρ_p and ρ_n. Decreasing this parameter has the opposite effect. Indeed, this parameter is used to calibrate the size of the FAs plaque without changing the relative equilibrium.

7. L is simply used as a switch, discussed in the main body of the paper.

8. τ controls the strength of the biasing potential field. The stronger the field the faster the nascent myofibrils align with each other.
